# Redox Dynamics of the Atmosphere and Oceans Induced by the Paleoproterozoic Snowball Earth Events

**DOI:** 10.1111/gbi.70040

**Published:** 2025-12-26

**Authors:** Mariko Harada, Yuna Miura, Yasuto Watanabe, Kazumi Ozaki

**Affiliations:** ^1^ Department of Earth and Planetary Sciences Institute of Science Tokyo Tokyo Japan; ^2^ Institute for Extra‐Cutting‐Edge Science and Technology Avant‐Garde Research (X‐Star), Japan Agency for Marine‐Earth Science and Technology (JAMSTEC) Kanagawa Japan; ^3^ Department of Environmental Science Toho University Chiba Japan; ^4^ Earth System Division National Institute for Environmental Studies Tsukuba Japan; ^5^ Earth‐Life Science Institute, Institute of Science Tokyo Tokyo Japan

## Abstract

The Paleoproterozoic Earth underwent profound environmental changes, including multiple severe glaciations and fluctuations in atmospheric oxygen (O_2_) levels. However, the precise relationship between O_2_ evolution and the glaciations remains unclear. Here, we use a biogeochemical cycle model involving carbon, phosphorus, sulfur, and oxygen to investigate the redox dynamics of the ocean—atmosphere system following the climatic transition to a super‐greenhouse state after deglaciation. Our stochastic analysis reveals that climatic recovery on a timescale of ~10^5^ years from elevated atmospheric CO_2_ levels (> 0.2 atm) triggers an extensive oxidation of the atmosphere and oceans over the subsequent 10^6^–10^7^ years, aligning with the large sulfur isotope anomaly in buried pyrite after the third Paleoproterozoic glaciation (~2.3 Ga). This finding suggests that the third glaciation represented an extensively glaciated, snowball state, which would have required massive accumulation of atmospheric CO_2_ for deglaciation. Variation in the boundary conditions regarding the global redox budget, as represented by high reductant fluxes, may explain the return of atmospheric O_2_ to Archean‐like levels following the first (Makganyene) glaciation, which is also considered a snowball Earth.

## 1 Introduction

The early Paleoproterozoic is a turning point in Earth's history, characterized by a transition from a reducing to an oxidizing atmosphere, known as the Great Oxidation Event (GOE), and repeated severe glaciations (Holland 2006; Lyons et al. 2014; Luo et al. 2022). Based on the multiple occurrences of glacial deposits in several localities (Evans et al. 1997; Kirschvink et al. 2000; Young 2002; Brasier et al. 2013), it has been suggested that there were at least three, possibly four global‐scale glacial periods during the Paleoproterozoic (Rasmussen et al. 2013; Gumsley et al. 2017; Luo et al. 2022). While lines of evidence indicate that the fluctuations of atmospheric O_2_ concentration occurred in association with the glaciations (Kirschvink et al. 2000; Bekker et al. 2004; Kopp et al. 2005; Sekine et al. 2010; Sekine, Tajika, et al. 2011; Sekine, Suzuki, et al. 2011; Goto et al. 2013; Luo et al. 2016; Poulton et al. 2021; Izon et al. 2022; Millikin et al. 2024), the causal relationship and mechanisms behind them remain obscure.

**FIGURE 1:**
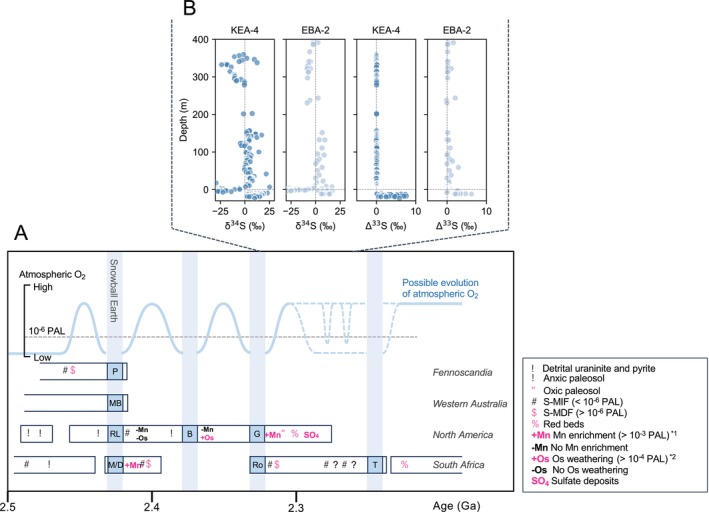
(A) Geological records and possible evolution of the atmospheric O_2_ level during the Paleoproterozoic. The geological data are sourced from compilations by previous studies (Luo et al. 2016; Gumsley et al. 2017), geochemical data (Sekine, Tajika, et al. 2011; Sekine, Suzuki, et al. 2011; Goto et al. 2013), and recent age constraints (Millikin et al. 2024). Symbols in magenta represent records suggesting atmospheric O_2_ concentrations greater than 10^−6^ PAL. The O_2_ level constraints of manganese (Mn) enrichment*^1^ and osmium (Os) weathering*^2^ are based on Sekine et al. (Sekine, Tajika, et al. 2011) and Sekine et al. (Sekine, Suzuki, et al. 2011), respectively. Blue vertical bars indicate periods of glaciation (B, Bruce; G, Gowganda; M/D, Makganeyne/Duitschland; MB, Meteorite Bore; P, Polisarka; RL, Ramsey Lake; Ro, Rooihoogte; T, Timeball Hill). (B) Sulfur isotope records (sulfide δ^34^S and S‐MIF) following the third glaciation are presented, with data from two distinct drilling cores in the Transvaal basin: KEA‐4 (Luo et al. 2016; Izon et al. 2022) and EBA‐2 (Poulton et al. 2021). The zero‐depth level represents the Rooihoogte–Timeball Hill boundary (Luo et al. 2016; Izon et al. 2022).

The disappearance of sulfur isotope mass‐independent fractionation (S‐MIF), which constrains the atmospheric O_2_ levels to > 10^−6^ present atmospheric level (PAL), has been considered one of the best proxies for the GOE (Farquhar et al. 2000; Pavlov and Kasting 2002; Zahnle et al. 2006; Catling and Zahnle 2020). However, the accumulated S‐MIF signals reveal a complex oxygenation history of the atmosphere during the Paleoproterozoic. One of the earliest examples of this complexity is recorded in the 2.43–2.44 Ga Polisarka diamictite in Fennoscandia, which correlates with the Makganyene diamictite in the Transvaal Supergroup (South Africa) and the Ramsey Lake diamictite in the Huronian Supergroup (North America) (Figure 1). These deposits represent the first (oldest) of the four Paleoproterozoic glaciations and are interpreted as evidence for a Snowball Earth event (Gumsley et al. 2017; Warke et al. 2020). The loss of an S‐MIF signal under the Polisarka diamictite in Fennoscandia (Warke et al. 2020) suggests that the increase in atmospheric O_2_ (> 10^−6^ PAL) preceded the first glaciation (Gumsley et al. 2017; Warke et al. 2020) (Figure 1). The occurrence of massive manganese (Mn) deposits above low‐latitude Makganyene glacial deposits has been interpreted as evidence for the strong oxidation of the atmosphere and oceans after the Snowball Earth event (Kirschvink et al. 2000; Kopp et al. 2005). However, this oxygenation was a temporal phenomenon and S‐MIF signals have been found in later formations deposited in both the Transvaal Supergroup in South Africa and the Huronian Supergroup in North America (Bekker et al. 2004; Papineau et al. 2007; Gumsley et al. 2017; Luo et al. 2022). Specifically, S‐MIF signals in the Huronian Supergroup suggest that atmospheric O_2_ levels returned to a low level (< 10^−6^ PAL) before the second (Bruce) glaciation (Papineau et al. 2007). The S‐MIF signals and the abundance of redox‐sensitive elements in sedimentary rocks suggest that O_2_ again increased to > 10^−6^ PAL after the second and third (Rooihoogte/Gowganda) glaciations (Papineau et al. 2007; Sekine et al. 2010; Sekine, Suzuki, et al. 2011; Sekine, Tajika, et al. 2011; Luo et al. 2016) (Figure 1).

In South Africa, the shift from S‐MIF to sulfur isotope mass dependent fractionation (S‐MDF) has been reported directly above the Rooihoogte glacial deposits (Luo et al. 2016, 2022; Izon et al. 2022) (Figure 1). Importantly, a large negative excursion in δ^34^S^b^_pyr_ (< −25‰) after the S‐MIF/S‐MDF transition suggests the buildup of sulfate (SO_4_^2−^) in the ocean, implying the expansion of highly oxygenated environments in the oceans (Luo et al. 2016). The thallium isotope ratio supports ocean oxygenation to the extent that Mn is oxidized following deglaciation (Ostrander et al. 2024). These records constrain the timing of the permanent oxidation above 10^−6^ PAL to ~2.32 Ga, shortly after the third glaciation (Luo et al. 2016). However, additional S‐MIF data complicate this picture, with sporadic reappearances of S‐MIF signals in the upper Rooihoogte Formation suggesting an age of ~2.2 Ga for permanent oxidation of the atmosphere (Poulton et al. 2021) (Figure 1). Such variations in the S‐MIF signals may reflect the recycling of older S‐MIF through oxidative weathering, the crustal memory effect (Reinhard et al. 2013; Philippot et al. 2018; Killingsworth et al. 2019), or short‐term local fluctuations in atmospheric oxygen levels, and this interpretation remains a subject of vigorous debate (Izon et al. 2022; Uveges et al. 2023). Despite accumulated geological records of S‐MIF and δ^34^S_pyr_ implying a causal link between glaciations and O_2_ increases, the exact timing of the ultimate shift from S‐MIF to S‐MDF, namely, the onset of permanent oxidation of the atmosphere, the GOE, and its connection to the glaciations, remains elusive.

To interpret these geological records, several theoretical studies have explored the relationship between climate events and the rise of atmospheric O_2_ (Harada et al. 2015; Garduno Ruiz et al. 2023, 2024). During a hard snowball Earth glaciation, defined as a climatic state in which the oceans are entirely covered by thick sea ice and open‐water areas are entirely absent, global surface temperatures drop to ~230 K. However, once sufficient atmospheric CO_2_ accumulates, rapid deglaciation occurs within ~10^3^ years (Hyde et al. 2000), followed by a transition to a strong greenhouse state (Tajika 2007). This climatic upheaval leads to an immediate increase in atmospheric O_2_ levels, driven by changes in water vapor levels and the production of oxidizing radicals (Garduno Ruiz et al. 2023, 2024). In addition, following 10^5^‐year‐scale climate recovery from the strong greenhouse state, the increases in O_2_‐producing photosynthesis in the nutrient‐enriched oceans cause a long‐term increase in atmospheric O_2_ levels (Harada et al. 2015). Notably, such perturbations in the carbon cycle likely caused substantial increases in O_2_ levels (Harada et al. 2015), which in turn elevated oceanic SO_4_^2−^ via the oxidative weathering of continental sulfide. However, the quantitative impact of these climatic variations on the sulfur cycle, including the oceanic SO_4_^2−^ concentration and δ^34^S, has not been thoroughly examined. Furthermore, the differing effects of transient versus permanent oxidation events on the sulfur cycle remain poorly understood.

**FIGURE 2:**
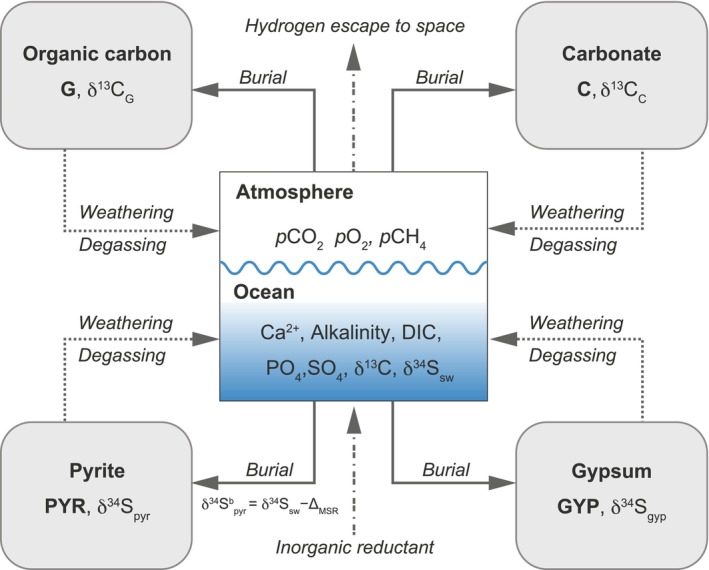
Schematic representation of the biogeochemical box model. Boxes represent reservoirs, while arrows indicate flux terms. The model captures the time evolution of major reservoirs and fluxes within the carbon, sulfur, oxygen, and phosphorus cycles in the atmosphere–ocean system, including a detailed representation of the ocean biogeochemistry and long‐term exchanges between the ocean–atmosphere system and the crust (e.g., sedimentary carbonate, organic carbon, gypsum, and pyrite). The carbon and sulfur isotope compositions (δ^13^C and δ^34^S) in each reservoir are also simulated based on variations in the carbon and sulfur cycles. DIC refers to dissolved inorganic carbon.

Here, we use a biogeochemical model of the carbon (C), phosphorus (P), sulfur (S), and O_2_ cycles to quantitatively evaluate the biogeochemical dynamics following deglaciation (Figure 2; Methods). We focus on simulating responses in the atmospheric O_2_ level, oceanic SO_4_^2−^ concentration, and δ^34^S^b^_pyr_ to a climate jump at the termination of the glaciation, aiming to constrain redox dynamics following the Paleoproterozoic glaciations.

## 2 Methods

### 2.1 Model Concept

The model developed for this study is an extension of a previous biogeochemical cycle model (Harada et al. 2015) that tracks the coupled C, P, and O_2_ cycles in the ocean–atmosphere system represented by three coupled boxes: the atmosphere, surface ocean, and deep ocean. The original model, which was previously applied to the aftermath of the Paleoproterozoic Makganyene Snowball glaciation, can be used to assess the effect of the postglacial greenhouse climate on the rate of continental weathering, nutrient (P) availability in the ocean, rate of O_2_‐production via photosynthesis, and atmospheric O_2_ level over geological timescales. We further enhance this model by incorporating a global biogeochemical S cycle, allowing us to simulate exchanges between oceanic SO_4_^2−^, sedimentary gypsum (CaSO_4_), and pyrite (FeS_2_). Our model also evaluates the δ^34^S values of the sulfur reservoirs, assuming that isotope fractionation occurs via microbial sulfate reduction (MSR), with the degree of fractionation (Δ_MSR_) being a function of the oceanic SO_4_^2−^ concentration (Habicht et al. 2002, 2005; Reinhard et al. 2013). These improvements enable a comprehensive estimation of the responses of the atmospheric O_2_ level and oceanic SO_4_^2−^ concentration to postglacial climate recovery, constrained by geological δ^34^S^b^_pyr_ records.

The model includes the atmosphere and ocean O_2_ (**O**), CO_2_ (**A**), and methane (CH_4_) (**M**), along with the oceanic phosphorus (**P**), sulfate (**S**), calcium (**Ca**), and alkalinity (**ALK**) reservoirs. Sedimentary reservoirs of carbon and sulfur were also included, that is, organic carbon (**G**), carbonate carbon (**C**), pyrite (**PYR**), and gypsum (**GYP**). We track the time evolution of these reservoirs by numerically integrating their coupled mass balance differential equations over time. The model also simulates the time evolution of the stable isotopic compositions of carbon and sulfur (δ^13^C and δ^34^S, respectively) within oceanic and sedimentary reservoirs. The global surface temperature is estimated using a zero‐dimensional energy balance model (Ozaki and Reinhard 2021), where the absorption of solar radiation is balanced by heat loss via outgoing longwave radiation under a given solar luminosity, atmospheric *p*CO_2_, and atmospheric *p*CH_4_ (Williams and Kasting 1997; Byrne and Goldblatt 2014).

The treatment of S cycling in this model is based on the COPSE framework (Bergman et al. 2004; Lenton et al. 2018) and does not explicitly calculate the MSR rates or dissolved H_2_S reservoirs. Similar to COPSE, the model parameterizes the rate of pyrite precipitation as a function of the oceanic SO_4_^2−^ concentration and the burial flux of organic carbon, thereby indirectly accounting for the effect of MSR on the precipitation rate. For simplicity, the reaction between SO_4_^2−^ and CH_4_ through the anaerobic oxidation of methane (AOM) is not considered. The subsequent section provides a detailed description of the major updates to the model implemented in this study. A further model description of the model is available in the Supporting Information: Table S1–S3.

### 2.2 Mass Balance Equations of Sulfur and Oxygen

The mass balances of SO_4_^2−^ in the surface (**S**_S_) and deep (**S**_D_) ocean boxes are expressed as follows:

(1) $$\frac{dS_{S}}{\mathrm{dt}}=F_{\text{vgyp}}+F_{\text{vpyr}}+F_{\text{wgyp}}+F_{\text{wpyr}}-F_{\text{bgyp}}-F_{\text{bpyr}}+F_{\mathrm{cir}\_S},$$

(2) $$\frac{dS_{D}}{\mathrm{dt}}=-F_{\mathrm{cir}\_S},$$

where *F* denotes the flux (in terms of mol yr.^−1^). Oceanic SO_4_^2−^ is supplied through degassing of continental gypsum (*F*_vgyp_) and pyrite (*F*_vpyr_) derived from volcanic and metamorphic sources, together with the weathering of continental gypsum (*F*_wgyp_) and pyrite (*F*_wpyr_), as described in detail in the next section. The removal of oceanic SO_4_^2−^ occurs via precipitation of abiotically formed sulfate evaporite (gypsum) and MSR, with MSR leading to the formation of sulfide minerals. In this study, we adopt the representation in the COPSE model for sulfate removal, assuming burial of gypsum (*F*_bgyp_) and pyrite (*F*_bpyr_) as sinks for oceanic SO_4_^2−^. We assume that all of these processes take place in the surface ocean box for simplicity. The surface and deep ocean boxes exchange sulfate via ocean circulation (*F*_cirS_). The fluxes are calculated at each time step, and both time‐dependent fluxes and reservoir sizes are reported. Where relevant, quasi‐steady states are identified by a sulfur budget balance.

The sedimentary reservoirs of gypsum (**GYP**) and pyrite (**PYR**) increase via the burial of sedimentary gypsum and pyrite (*F*_bgyp_ and *F*_bpyr_) and decrease via degassing (*F*_vgyp_ and *F*_vpyr_) and weathering (*F*_wgyp_ and *F*_wpyr_). The mass balance equations are as follows:

(3) $$\frac{\text{dGYP}}{\mathrm{dt}}=F_{\text{bgyp}}-F_{\text{vgyp}}-F_{\text{wgyp}},$$

(4) $$\frac{\text{dPYR}}{\mathrm{dt}}=F_{\text{bpyr}}-F_{\text{vpyr}}-F_{\text{wpyr}}.$$

The formation and decomposition of reduced sulfur (pyrite) serve as a long‐term source and sink of O_2_. Therefore, we incorporated the pyrite degassing, weathering, and burial fluxes into the O_2_ mass balance of the Harada et al. (2015) model.

The mass balance of O_2_ reservoirs (O_2_ in the atmosphere–surface ocean: **O**_**AS**_, and O_2_ in the deep ocean: **O**_**D**_) is expressed as follows:

(5) $$\begin{array}{c} \frac{dO_{\mathrm{AS}}}{\mathrm{dt}}=F_{\mathrm{oph}}-F_{\mathrm{dgS}\_O}-F_{\mathrm{voc}}-F_{\mathrm{wo}\_O}+2\left(F_{\text{bpyr}}-F_{\text{vpyr}}-F_{\text{wpyr}}\right)\\ \;-F_{\mathrm{esc}}-F_{\mathrm{oxi}\_O}+F_{\mathrm{cir}\_O}, \end{array}$$

(6) $$\frac{dO_{D}}{\mathrm{dt}}=-F_{\mathrm{dgD}\_O}-F_{\mathrm{cir}\_O}.$$

O_2_ in the atmosphere–surface ocean is generated via oxygenic photosynthesis (*F*_oph_) and is consumed by the aerobic respiration of organic matter (*F*_dgS_O_), the decomposition of terrestrial organic carbon via degassing (*F*_voc_) and oxidative weathering (*F*_wo_O_), photochemical methane oxidation in the atmosphere (*F*_oxi_O_), and hydrogen escape at the top of the atmosphere to space (*F*_esc_). In the deep ocean box, oxygen is supplied via exchange between the surface and deep oceans (*F*_cir_O_) and is consumed by the decomposition of particulate organic matter (*F*_dgD_O_). We incorporate the impact of the formation and decomposition of reduced sulfur: the net increase in the pyrite reservoir burial (*F*_bpyr_) minus degassing (*F*_vpyr_) and weathering (*F*_wpyr_) led to an increase in O_2_ in the atmosphere and ocean. The atmosphere and surface ocean reservoirs are treated as a single box at each time step, with subsequent separation of the mass of the components into the two boxes (i.e., an atmosphere box and a surface ocean box), assuming the temperature‐dependent equilibrium gas exchange between the atmosphere and the surface ocean, based on the model‐calculated surface temperature. The oxidative weathering of continental pyrite can also influence the carbon cycle through the production of acids that dissolve continental carbonates (Kölling et al. 2019). In this study, this process is not incorporated for simplicity because of the uncertainty in how extensively the carbonate weathering is affected.

### 2.3 Degassing and Weathering of Gypsum and Pyrite

The release of sulfur into the atmosphere and ocean system through volcanic and metamorphic degassing is assumed to be proportional to the size of the sedimentary reservoirs. This assumption is supported by geochemical analyses showing that the global mantle sulfur degassing flux is much smaller than the arc‐related flux (Kagoshima et al. 2015). The degassing of gypsum and pyrite is expressed as,

(7) $$F_{\text{vgyp}}=F_{\text{vgyp}}^{*}\frac{\mathrm{GYP}}{{\mathrm{GYP}}^{*}},$$

(8) $$F_{\text{vgyp}}=F_{\text{vgyp}}^{*}\frac{\mathrm{PYR}}{{\mathrm{PYR}}^{*}}.$$

where * represents a reference value. In this study, we assume GYP* = 150 × 10^18^ mol and PYR* = 250 × 10^18^ mol as reference values. The constants *F*_vgyp_* and *F*_vpyr_* denote the present fluxes of gypsum and pyrite degassing, respectively, with *F*_vgyp_* = 0.5 Tmol S yr.^−1^ and *F*_vpyr_* = 0.3 Tmol S yr.^−1^ assumed in this study. These values fall within the ranges of published estimates (Ozaki et al. 2022); the total modern volcanic degassing is 0.3–3 Tmol S yr.^−1^ (Walker and Brimblecombe 1985; Raiswell and Canfield 2012; Kagoshima et al. 2015; Catling and Kasting 2017) and the present reservoir sizes for **GYP** and **PYR** are 77–300 × 10^18^ mol and 155–300 × 10^18^ mol, respectively (Holser et al. 1989; Kump and Byrne 1989; Lasaga 1989; Sleep 2005; Berner 2006; Bottrell and Newton 2006; Yaroshevsky 2006; Schlesinger and Bernhardt 2020).

The release of sulfur into the ocean–atmosphere system through weathering is also assumed to be proportional to the size of the sedimentary reservoirs. The weathering flux of gypsum is assumed to be proportional to the carbonate weathering flux (*F*_*wc*_) normalized by the present value (*F*_wc_*), whereas the rate of pyrite oxidative weathering is assumed to be dependent on the atmospheric *p*O_2_ normalized by the present oxygen concentration (*p*O_2_*). In this study, we adopt a Monod‐type relationship between the oxidative weathering rate and atmospheric *p*O_2_, which reflects the fact that under highly oxygenated conditions, the rate of oxidative weathering reaches its maximum, as constrained by the erosion rate (Ozaki et al. 2022).

(9) $$F_{\text{wgyp}}=F_{\text{wgyp}}^{*}\frac{\mathrm{GYP}}{{\mathrm{GYP}}^{*}}\frac{F_{\mathrm{wc}}}{F_{\mathrm{wc}}^{*}},$$

(10) $$F_{\text{wpyr}}=F_{\text{wpyr}}^{*}f_{a}\;c_{\text{pyrw}}\left\{\frac{\left(\frac{pO_{2}}{{\mathrm{pO}}_{2}^{*}}\right)}{\left(\frac{pO_{2}}{{\mathrm{pO}}_{2}^{*}}\right)+k_{\text{pyrw}}}\right\}\frac{\mathrm{PYR}}{{\mathrm{PYR}}^{*}}.$$

The constants *F*_wgyp_* and *F*_wpyr_* represent the present rates of gypsum and pyrite weathering, where we assume 1.6 Tmol S yr.^−1^ and 1.3 Tmol S yr.^−1^, respectively. The constant *f*_a_ denotes the relative continental area (*f*_a_ = 1 at present), for which we assume a Paleoproterozoic value of 0.48. The constants *k*_pyrw_ and *c*_pyrw_ correspond to the half‐saturation constant (*k*_pyrw_ = 0.017) and the normalized constants (*c*_pyrw_ = 1.017), respectively. The total modern river S input (*F*_riv.S_* = *F*_wgyp_* + *F*_wpyr_*) is 2.9 Tmol S yr.^−1^. These values are roughly consistent with observation‐based estimates: 1.5 ± 0.2 Tmol S yr.^−1^ and 1.3 ± 0.2 Tmol S yr.^−1^ for modern weathering rates of gypsum and pyrite (Burke et al. 2018), respectively, and a total river S input of 2.6 ± 0.6 Tmol S yr.^−1^ (Raiswell and Canfield 2012).

### 2.4 Burial of Gypsum and Pyrite

Burial rates of gypsum (*F*_bgyp_) are assumed to be proportional to the SO_4_^2−^ and Ca^2+^ concentrations in the surface ocean ([Ca] and [SO_4_], respectively) (Berner 2004).

(11) $$F_{\text{bgyp}}=F_{\text{bgyp}}^{*}\frac{\left[\mathrm{Ca}\right]}{{\left[\mathrm{Ca}\right]}^{*}}\frac{\left[{\mathrm{SO}}_{4}\right]}{{\left[{\mathrm{SO}}_{4}\right]}^{*}}.$$

The constants [Ca]* and [SO_4_]* denote the present values of the sulfate and calcium concentrations. The constant *F*_bgyp_* is a standard value of the gypsum burial rate that balances with the sum of gypsum weathering and degassing rates at the present steady state (*F*_bgyp_* = *F*_vgyp_* + *F*_wgyp_* = 2.1 Tmol S yr.^−1^).

The burial rate of sedimentary pyrite (*F*_bpyr_) is defined as a function of the oceanic SO_4_^2−^ concentration ([SO_4_]), the atmospheric oxygen concentration (*p*O_2_), and the burial rate of sedimentary organic carbon (*F*_bo_) as follows: (Bergman et al. 2004).

(12) $$F_{\text{bpyr}}=F_{\text{bpyr}}^{*}\frac{\left[{\mathrm{SO}}_{4}\right]}{{\left[{\mathrm{SO}}_{4}\right]}^{*}}\frac{{\mathrm{pO}}_{2}^{*}}{pO_{2}}\frac{F_{\mathrm{bo}}}{F_{\mathrm{bo}}^{*}},$$

where the constants *F*_bpyr_*, [SO_4_]*, pO_2_*, and *F*_bo_* represent the modern values of each parameter. The modern rate of sedimentary pyrite burial is therefore estimated to be *F*_bpyr_* = *F*_vpyr_* + *F*_vpyr_* = 1.8 Tmol S yr.^−1^ in this model.

### 2.5 Isotope Fractionation via Microbial Sulfate Reduction

Studies have reported that the magnitude of sulfur isotope fractionation varies with both the SO_4_^2−^ concentration and the cell‐specific sulfate reduction rate (csSRR), with a large fractionation occurring at high SO_4_^2−^concentration and low csSRR and fractionation becoming smaller at low SO_4_^2−^ concentration and high csSRR (Habicht et al. 2002, 2005; Bradley et al. 2016; Sim et al. 2023). We adopt a Michaelis–Menten‐type function to express the relationship between the magnitude of sulfur isotope fractionation (Δ_MSR_) and the SO_4_^2−^ concentration (Habicht et al. 2002, 2005; Reinhard et al. 2013):

(13) $${∆}_{\mathrm{MSR}}={∆}_{\max}\frac{\left[{\mathrm{SO}}_{4}\right]}{\left[{\mathrm{SO}}_{4}\right]+K_{\mathrm{MSR}}},$$

where Δ_max_ and *K*_MSR_ represent the maximum value of the sulfur isotope fractionation and the sulfate half‐saturation concentration, respectively. The magnitude of the sulfur isotope fractionation is inversely correlated to csSRR (Bradley et al. 2016); here, this effect is incorporated into the value of Δ_max_. The values of Δ_max_ and *K*_MSR_ vary among strains; therefore, it is difficult to determine specific values for the Proterozoic global average. In the nominal simulation, we adopted *K*_MSR_ = 200 μM; this value is roughly consistent with 363 μM of *Archaeoglobus fulgidus* strain Z (Habicht et al. 2005) and the apparent relationships between Δ_MSR_ and the sulfate concentration reported for other strains (Figure S5). We assumed Δ_max_ = 40.0‰ for the nominal simulation to reproduce the present isotopic fractionation between sulfate and sulfide under the present boundary conditions. To consider the effect of the uncertainty, Monte Carlo simulations were conducted varying both Δ_max_ and *K*_MSR_. Although temperature may influence cellular‐level sulfur isotope fractionation, experimental data suggest that enzymatic isotope effects show little temperature dependence (Sim et al. 2023). Accordingly, the model assumes biologically controlled but temperature‐independent sulfur isotope fractionation.

### 2.6 Redox‐Dependent Phosphorus Burial

The burial rate of P (*F*_bo_P_) in the sediment is calculated from the marine organic carbon burial rate (*F*_bo_) and the C/P burial ratio (*CP*_*sea*_):

(14) $$F_{\mathrm{bo}\_P}=\frac{F_{\mathrm{bo}}}{{\mathrm{CP}}_{\mathrm{sea}}},$$

where *CP*_*sea*_ depends on ocean anoxia, as described by Lenton et al. (Lenton et al. 2018):

(15) $${\mathrm{CP}}_{\mathrm{sea}}=\frac{k_{\text{anox}}\;k_{\text{oxic}}}{\left(1-\text{anox}\right)k_{\text{anox}}+\text{anox}\;k_{\text{oxic}}}.$$

here, *k*_*oxic*_ and *k*_*anox*_ represent the C/P burial ratio of organic matter under oxic and anoxic conditions, respectively. We follow COPSE (Bergman et al. 2004) to estimate the anoxic fraction of the ocean (*anox*):

(16) $$a\mathrm{nox}=\max\left(1-k_{1}\frac{pO_{2}}{{\mathrm{pO}}_{2}^{*}}\frac{F_{\mathrm{pp}}^{*}}{F_{\mathrm{pp}}},0\right),$$

where *k*_1_ = 0.997527 represents the oxic fraction in the present‐day ocean (Lenton et al. 2018) and *F*_pp_* and *F*_pp_ are the present and simulated net primary productivities, respectively. In our model, *CP_sea_* is defined as the ratio between the buried organic carbon (C_org_) and the biologically reactive P (P_reac_), with P_reac_ being the sum of the sedimentary organic P (P_org_), Fe‐bound P (P_Fe_), and Ca‐bound P (P_Ca_) (Ozaki et al. 2022):

(17) $${\mathrm{CP}}_{\mathrm{sea}}=C_{\mathrm{org}}/P_{\text{reac}}=\frac{\text{Marine}\;C_{\mathrm{org}}\text{burial rate}}{\text{Marine}\;P_{\text{reac}}\text{burial rate}},$$

(18) $$P_{\text{reac}}=P_{\mathrm{org}}+P_{\mathrm{Fe}}+P_{\mathrm{Ca}}.$$

In Equation (15), we adopt *k*_anox_ = 200 and *k*_oxic_ = 60 as the *CP*_*sea*_ value for anoxic and oxic oceans, respectively, based on the estimations derived from various C_org_/P_reac_ observations of modern sediments (Algeo and Ingall 2007), because quantitative constraints from Archean and Paleoproterozoic sediments remain uncertain. The riverine P input rate is defined to balance *F*_bo_P_; thus, the evolution of the continental phosphorus reservoir (e.g., Cox et al. 2018; Walton et al. 2023) is not explicitly considered. However, since our analysis focuses on the system's response to postglacial climate perturbations from a given steady state, the uncertainty in the absolute riverine *P* flux does not significantly affect the overarching conclusions.

### 2.7 Initial and Boundary Conditions

We initiate the simulation from a low steady‐state atmospheric O_2_ level (i.e., ~10^−6^ PAL), which corresponds to a low branch of a stable steady state of atmospheric O_2_ (Goldblatt et al. 2006; Harada et al. 2015), and elevated initial atmospheric *p*CO_2_ levels (*p*CO_2_^init^). Prior to each simulation, spin‐up calculations were performed for ~10^8^ years to achieve steady states under the Paleoproterozoic low‐O_2_ boundary conditions, with the initial size of total continental sulfur being 400 × 10^18^ mol (**PYR** = 400 × 10^18^ and **GYP** ~ 0 mol, respectively). We assume reduced solar luminosity (~83% of the present value) and low continental weatherability (~12% of the present‐day value) for the Paleoproterozoic boundary conditions, under which the steady‐state atmospheric *p*CO_2_ level is ~0.087 atm. We mainly focus on the post‐third‐glaciation interval (tens of Myr); thus, luminosity is treated as constant, with its value calculated based on a solar evolution model of Gough (1981). The low continental weatherability adopted here is derived from estimates of weak soil biological activity due to the absence of land plants and the limited continental area (Drever and Zobrist 1992; Krissansen‐Totton et al. 2018). The values adopted in this study (Table S3) fall within the uncertainty range of previous estimates (Krissansen‐Totton et al. 2018). Sensitivity experiments (Harada et al. 2015) show that reasonable variations in weatherability do not significantly alter the qualitative O_2_ trajectories. Simulations were then started with increased atmospheric *p*CO_2_ values. Following deglaciation, the post‐snowball Earth ocean is thought to have been salinity‐stratified; however, such stratification is estimated to collapse within a relatively short timescale on the order of 10^3^ years (Ramme and Marotzke 2020). Therefore, we assume that the initial condition represents a fully deglaciated ocean without persistent salinity stratification. Given that the maximum estimate of the atmospheric *p*CO_2_ level required to terminate a Paleoproterozoic snowball Earth glaciation is ~0.7 atm (Tajika 2003), with potential uncertainties due to lower albedo during glaciations (Abbot and Pierrehumbert 2010), we vary *p*CO_2_^init^ from 0.1 to 0.7 atm. The initial values of the carbon reservoir (**A**) and carbon isotope ratio (δ^13^C) in the atmosphere–surface ocean box were determined according to the initial atmospheric *p*CO_2_. In addition to the conditions described above, initial values for all the parameters are taken from steady‐state values.

Boundary conditions affecting the O_2_ mass balance (e.g., the input rate of reducing materials, degassing rate of CO_2_, and continental land area) are important factors controlling the steady state of atmospheric O_2_ level and the occurrence of permanent oxidation (Kadoya et al. 2020; Watanabe and Tajika 2021; Wogan et al. 2022; Garduno Ruiz et al. 2023, 2024). In the present study, to explore the behavior of the redox dynamics and its relationship with geological records under different boundary conditions, we conduct sensitivity studies varying the rate of reductant input from the Earth's interior (*F*_red_ = 0.3 and 0.075 Tmol O_2_ equiv. yr.^−1^), using values based on pre‐GOE iron (Fe) deposition rates and post‐GOE hydrothermal Fe inputs, as estimated in a previous model (Goldblatt et al. 2006). We then perform Monte Carlo simulations using a wide range of potential parameter values, subsampling model runs that roughly reproduce the δ^34^S_pyr_ records. Via a series of sensitivity experiments, we constrain the conditions that can explain the large negative δ^34^S^b^_pyr_ anomaly after the third glaciation and explore how biogeochemical responses differ when permanent atmospheric oxygenation is achieved and when the atmospheric O_2_ levels drop to Archean‐levels (< 10^−6^ PAL).

## 3 Results and Discussion

### 3.1 Large Climatic Perturbations Needed to Explain the Large Negative δ^34^S_pyr_ Anomaly

**FIGURE 3:**
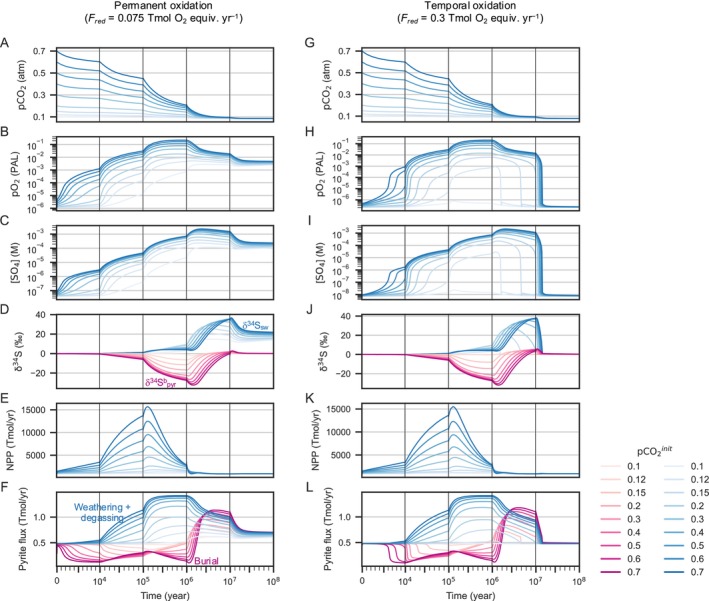
Time evolution of selected parameters in nominal runs varying with the initial value of the atmospheric *p*CO_2_ under the permanent oxidation scenario (*F*_red_ = 0.075 Tmol O_2_ equiv. yr.^−1^; A−F) and the temporal oxidation scenario (*F*_red_ = 0.3 Tmol O_2_ equiv. yr.^−1^; G−L). (A, G) Partial pressure of atmospheric CO_2_; (B, H) partial pressure of atmospheric O_2_; (C, I) oceanic sulfate concentration ([SO_4_^2−^]); (D, J) sulfur isotope compositions of seawater (δ^34^S_sw_, blue) and buried pyrite (δ^34^S^b^_pyr_, purple); (E, K) oceanic net primary production (NPP); and (F, L) pyrite S flux (blue lines: Sum of weathering and degassing fluxes, purple lines: Burial flux).

The results indicate that higher *p*CO_2_^init^ values result in larger increases in atmospheric O_2_ and oceanic SO_4_^2−^ (Figure 3A–C), as well as larger anomalies in the sulfur isotope composition of seawater (δ^34^S_sw_) and buried pyrite (δ^34^S^b^_pyr_) (Figure 3D). Note that even though small perturbations (*p*CO_2_^init^ = 0.1 atm) can trigger atmospheric oxidation, the simulated δ^34^S^b^_pyr_ is insufficient to reproduce the amplitude of the observed negative excursion, suggesting that a large climatic perturbation is required to explain the large (< −25‰) negative δ^34^S^b^_pyr_ anomaly.

Indeed, large perturbation scenarios (e.g., *p*CO_2_^init^ = 0.7 atm) cause negative δ^34^S^b^_pyr_ anomalies consistent with geologic records. Such scenarios show a hot climate (*T* ~ 330 K) continuing for ~10^5^ years (Figures 3A, S1). The greenhouse climate enhances chemical weathering on land, which leads to the transport of phosphorus from the continents to the ocean (Figure S1). This results in a marked increase in atmospheric O_2_ (Figure 3B) by promoting oceanic biological productivity (Figure 3E) and the following burial of organic matter in sediments, a major O_2_ source over geological timescales. High *p*CO_2_^init^ causes an overshoot, that is, a temporary spike in atmospheric O_2_. Notably, the amplitude of the overshoot depends strongly on *p*CO_2_^init^. In our nominal simulation, *p*CO_2_^init^ > 0.15 atm is required for the overshoot of an atmospheric O_2_ exceeding 0.01 PAL, while *p*CO_2_^init^ > 0.5 atm is necessary to induce an overshoot of > 0.1 PAL (Figure 3B). The increase in atmospheric O_2_ leads to a gradual increase in the oceanic SO_4_^2−^ concentration (Figure 3C) resulting from the combined effect of the enhanced riverine input of SO_4_^2−^ via the oxidative weathering of pyrite and inhibited pyrite burial in the oxygenated oceans (Figure 3F). More specifically, SO_4_^2−^ levels increase to > 1 mM for *p*CO_2_^init^ > 0.5 atm. The buildup of SO_4_^2−^ in the ocean enhances the isotopic fractionation via MSR, leading to a large negative excursion of buried pyrite (< −25‰) within ~10^6^ years following deglaciation (Figure 3F).

On longer timescales, the atmospheric O_2_ decreases as a result of O_2_ consumption via the oxidative weathering of sedimentary pyrite and organic carbon (Figures 3F, S1). Deoxygenation of the ocean gives rise to a decrease in SO_4_^2−^ by enhancing MSR and the subsequent burial of pyrite (Figure 3F). The removal of isotopically light S via pyrite burial leaves heavy S in the ocean, resulting in positive δ^34^S_sw_ and δ^34^S^b^_pyr_ anomalies (~+ 20‰–30‰ and ~+5‰, respectively). After ~10^8^ years, the system reaches a quasi‐steady state that is different from the initial state, where the atmospheric O_2_ and oceanic SO_4_^2−^ levels and δ^34^S_sw_ are high (*p*O_2_ > 10^−3^ PAL, SO_4_^2−^ > 0.1 mM, and δ^34^S_sw_ = +15‰ − 20‰). The simulated quasi‐steady‐state values roughly match independent geological and numerical constraints on atmospheric O_2_ seawater SO_4_^2−^ concentrations, and δ^34^S datasets (Planavsky et al. 2012, 2014; Luo et al. 2015; Cole et al. 2016; Tang et al. 2016; Hardisty et al. 2017; Fakhraee et al. 2024).

### 3.2 Biogeochemical Dynamics With High External Input of Reductants

We conducted additional experiments (Figures 3G–L, S2) assuming a higher excess input of reductants from Earth's interior (*F*_red_) of 0.3 Tmol O_2_ equiv. yr.^−1^, which falls within the estimated range prior to the GOE (Goldblatt et al. 2006). Under such conditions, the atmospheric O_2_ has a single steady‐state value of ~10^−7^ PAL in our model. Accordingly, permanent oxidation of the atmosphere is not reached in these scenarios, regardless of the assumed climatic perturbation (Figure 3H). On timescales of < 10^7^ years, the time evolutions of the atmospheric O_2_ (Figure 3H) and oceanic SO_4_^2−^ (Figure 3I) are nearly identical to those in Figure 3B,C. Consequently, the sulfur fluxes and δ^34^S values also exhibit similar temporal variations: a large negative excursion of δ^34^S^b^_pyr_ followed by positive excursions of δ^34^S_sw_ and δ^34^S^b^_pyr_ (Figure 3J). However, on longer timescales (~10^7^ years), the atmospheric O_2_ and oceanic SO_4_^2−^ concentrations drop toward low steady‐state levels. This precludes sulfur isotope fractionation between sulfides and the seawater, with the sulfur isotope ratio between the two stabilizing around 0‰ (Figure 3J). Within several 10^7^ years, the system returns to the initial steady state characterized by low atmospheric O_2_ and oceanic SO_4_^2−^ levels (*p*O_2_ < 10^−6^ PAL and SO_4_^2−^ ~ 10^−5^ mM, respectively), and δ^34^S_sw_ = 0‰, contrasting with the outcomes of the low‐*F*_red_ experiment. The results of the sensitivity tests suggest that perturbations in the carbon cycle primarily drive temporal variations in δ ^34^S^b^_pyr_, while the boundary conditions influence the oceanic SO_4_^2−^ concentration and δ^34^S_sw_, through controlling the achieved steady‐state levels of atmospheric O_2_.

### 3.3 Conditions for the δ^34^S^b^_pyr_ Anomaly After the Third Glaciation

To assess the uncertainty of the model and to identify the conditions that account for the negative δ^34^S^b^_pyr_ anomaly observed after the third Paleoproterozoic glaciation, we performed Monte Carlo simulations in which we randomly sampled from a wide range of potential values for the model parameters. The parameters subject to sampling included *p*CO_2_^init^, the initial amount of sedimentary pyrite (PYR^init^), and both the half‐saturation constant (*K*_MSR_) and the maximum value (Δ_max_) for sulfur isotope fractionation via MSR (Table 1). The ranges of *K*_MSR_ and Δ_max_ were constrained to be consistent with laboratory data on sulfate‐reducing microorganisms (Sim et al. 2023). The initial *p*CO_2_ (*p*CO_2_^init^) was varied from 0.08 to 0.8 atm to encompass both the CO₂ level required for hard Snowball Earth deglaciation (~0.7 atm; Tajika 2003) and the equilibrium value estimated in this study (~0.087 atm). The initial pyrite reservoir (PYR^init^) was set between 50 and 500 × 10^18^ mol to cover the estimated range of the present‐day crustal pyrite reservoir (155–300 × 10^18^ mol; see Methods). We repeatedly ran the model (*N* = 10,000), and in each simulation the minimum value for δ^34^S^b^_pyr_ (δ^34^S^b,min^_pyr_) throughout the 10^8^‐year temporal variation was analyzed. Of the 10,000 runs, we subsampled model runs that roughly reproduced the range of the large negative anomaly (−35‰ < δ^34^S^b,min^_pyr_ < −25‰) recorded in the geological records (Luo et al. 2016).

**TABLE 1:** Range of values of the constants included in the Monte Carlo simulations.

	Range of values	Sampling	Nominal
*p*CO_2_^init^ (atm)	0.08–0.8	(uniform)	0.1–0.7 (varying)
PYR^init^ (10^18^ mol)	50–500	(uniform)	400
*K*_MSR_ (mM)	2.0 × 10^−2^ − 2.0	(log uniform)	2.0 × 10^−1^
Δ_max_ (‰)	10–60	(uniform)	40

**FIGURE 4:**
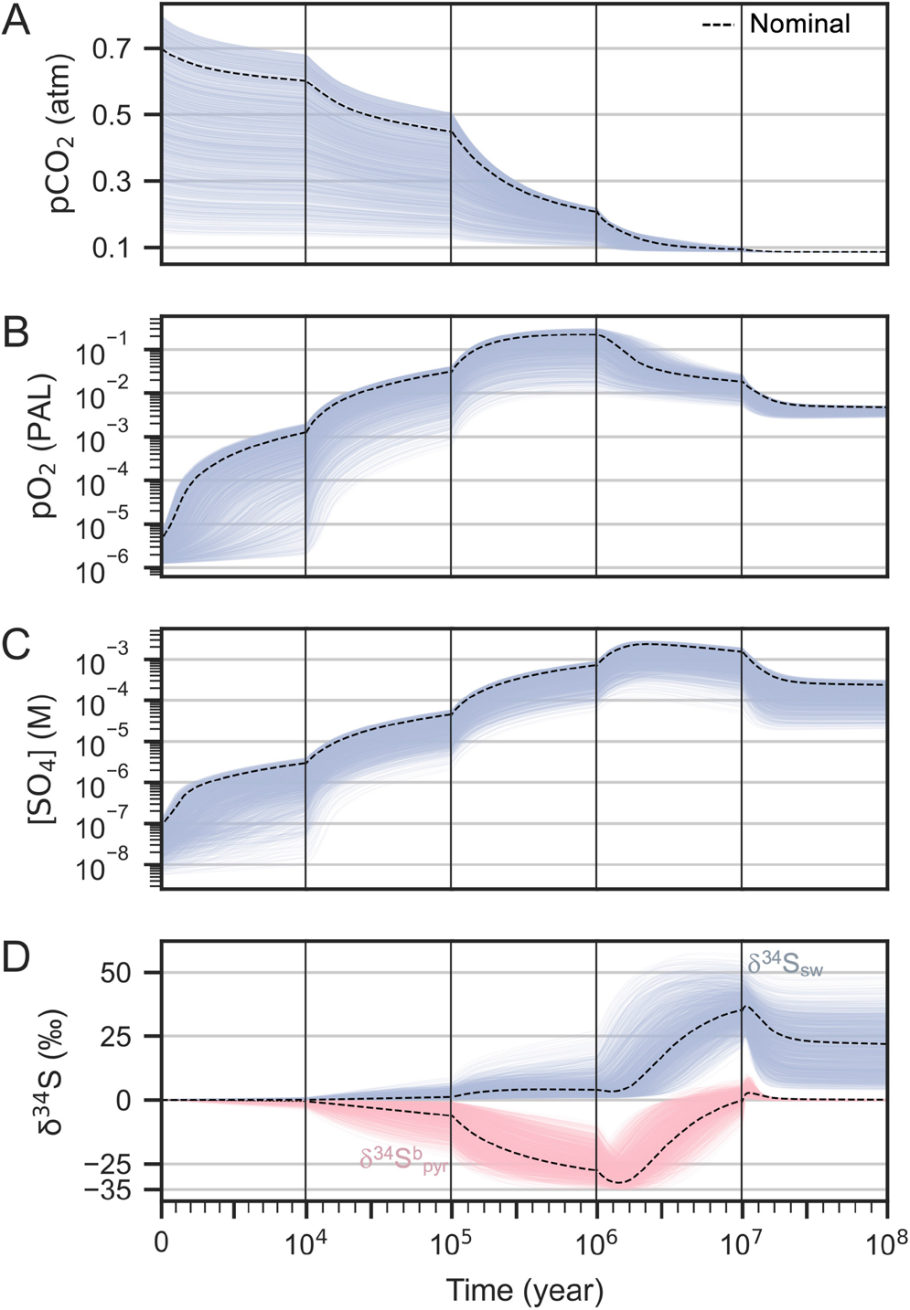
Output of subsampled runs (−35‰ < δ^34^S^b,min^_pyr_ < −25‰) out of 10,000 Monte Carlo simulations (*N* = 1945). (A) Partial pressure of atmospheric CO_2_; (B) partial pressure of atmospheric O_2_; (C) oceanic sulfate concentration ([SO_4_^2−^]); and (D) sulfur isotopes of seawater (δ^34^S_sw_, blue) and buried pyrite (δ^34^S^b^_pyr_, pink). Black dotted lines indicate the output of the nominal run with *p*CO_2_^init^ = 0.7 atm and *F*_red_ = 0.075 Tmol O_2_ equiv. yr.^−1^ (Figure 3A–D).

**FIGURE 5:**
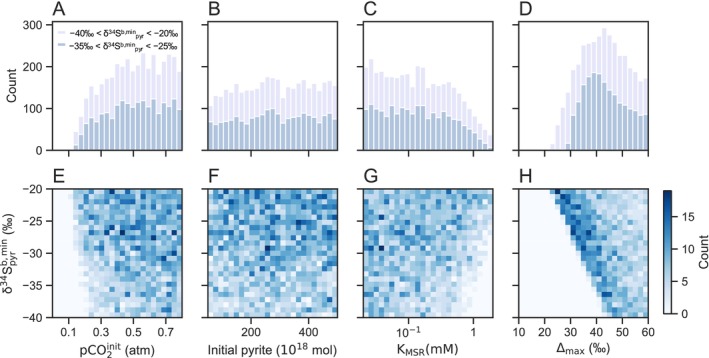
Histograms of accepted runs out of 10,000 Monte Carlo simulations. Counts of subsampled runs as functions of (A) *p*CO_2_^init^, (B) initial pyrite (PYR^init^), (C) *K*_MSR_, and (D) Δ_max_. Light gray histograms indicate runs with −40‰ < δ^34^S^b,min^_pyr_ < −20‰ (*N* = 3764), and dark gray histograms indicate runs with −35‰ < δ^34^S^b,min^_pyr_ < −25‰ (*N* = 1945). Counts of runs as functions of the δ^34^S^b,min^_pyr_ values and (E) *p*CO_2_^init^, (F) initial pyrite (PYR^init^), (G) *K*_MSR_, and (H) Δ_max_.

The results of all successful runs (*N* = 1945) indicate that at least 0.14 atm of *p*CO_2_^init^ is required to induce the negative anomaly observed in the geological records, even when accounting for large uncertainties in the model parameters (Figures 4A and 5A). When applying more conservative constraints (−40‰ < δ^34^S^b,min^_pyr_ < −20‰), 3764 runs were accepted; at least 0.12 atm of *p*CO_2_^init^ is required to satisfy these constraints (Figure 5A). These *p*CO_2_^init^ values exceed the equilibrium *p*CO_2_ level in this model by 62% and 38%, respectively. In the accepted runs, the atmospheric O_2_ levels reached up to 10^−2^ − 0.2 PAL and oceanic SO_4_^2−^ concentrations increased to values from a few hundred μM to 1 mM (Figure 4B,C). Consistent with the nominal experiments (Figure 3), the Monte Carlo results indicate that large fluctuations in the carbon cycle, driven by high CO₂ concentrations, are necessary for the overshoot in atmospheric O_2_ levels and oceanic SO_4_^2−^ concentrations, as well as the development of negative δ^34^S^b^_pyr_ anomalies.

Analyses of the relationship between δ^34^S^b,min^_pyr_ and each parameter reveal a strong dependence of δ^34^S^b,min^_pyr_ on *p*CO_2_^init^, *K*_MSR_, and Δ_max_. Within the entire range of *p*CO_2_^init^, achieving lower δ^34^S^b,min^_pyr_ requires lower *K*_MSR_ and higher Δ_max_ (Figures 5C,D, S3). We found that under small climatic perturbations, a negative anomaly of −35‰ < δ^34^S^b,min^_pyr_ < −25‰ occurs only for a limited combination of *K*_MSR_ and Δ_max_ values (Figures S3, S4). Notably, the runs with *p*CO_2_^init^ > 0.2 atm account for ~97% of the total accepted runs, whereas very few runs were accepted for *p*CO_2_^init^ < 0.2 atm (*N* = 65); these low‐*p*CO₂ cases were characterized by low *K*_MSR_ (< 0.15 mM) and large Δ_max_ (> 44‰) values (Figure S4). Strains that meet both values are rare among known extant organisms (Figure S5). As shown in Figure S5, experimental data display large variability in sulfur isotope fractionation at fixed sulfate concentrations, likely reflecting variations in csSRR. Our Monte Carlo ensemble captures this variability through broad sampling of Δ_max_, excluding high csSRR, low‐Δ_max_ cases. Although further constraining *K*_MSR_ and Δ_max_ for both extant and ancient organisms is necessary for more accurate determinations of the possible range of *p*CO_2_^init^, we can conclude that a *p*CO_2_^init^ value greater than 0.2 atm (exceeding the equilibrium *p*CO_2_ by 130%) is typically required to trigger a large negative δ^34^S^b^_pyr_ anomaly below −25‰.

Nutrient competition between oxygenic and anoxygenic primary producers (e.g., Jones et al. 2015; Ozaki et al. 2019; Watanabe et al. 2023) could affect transient O_2_ and sulfate dynamics after glaciation. Notably, Fe‐oxidizing phototrophs sustained by hydrothermal Fe^2+^ may compete for phosphate depending on the Fe/P ratio of upwelled deep water (Jones et al. 2015; Ozaki et al. 2019). Under such competition, higher *p*CO_2_^init^ would be required to reproduce the observed δ^34^S^b^_pyr_ anomaly. The effect of the ocean anoxia on the C/P burial ratio adopted here is based on the C_org_/P_reac_ ratio of sulfidic conditions (Algeo and Ingall 2007). However, in the Proterozoic ferruginous ocean, C_org_/P_reac_ may have been lower than that under sulfidic conditions because *P*_reac_ effectively bonded with Fe (Canfield et al. 2020). Accordingly, we also explored the option of a fixed burial ratio under low C_org_/P_reac_ conditions (*CP_sea_* = 60; Figures S6, S7). The results suggest that the assumption concerning C_org_/P_reac_ does not strongly alter our conclusions.

### 3.4 Implications for Geological Records

The large amount of atmospheric CO_2_ required by our model suggests that the third Paleoproterozoic glaciation represented an extensively glaciated, snowball state that necessitated a substantial CO_2_ buildup for deglaciation. For example, Tajika (2003) estimates that ~0.7 atm of CO_2_ would be necessary to melt a hard snowball Earth under early Paleoproterozoic conditions, although this value could be somewhat lower because of the altered albedo values during glaciations (Abbot and Pierrehumbert 2010). Deglaciation from a soft snowball Earth, which represents a severe but not globally frozen state with unfrozen equatorial regions, would also require atmospheric CO_2_ accumulation, but the amount should be significantly less than that for a hard snowball Earth (Crowley et al. 2001). Although our model cannot differentiate between hard and soft Snowball Earth states, the minimum atmospheric CO_2_ concentration of 0.2 atm indicated by our simulations is consistent with an extensively glaciated, snowball state.

An alternative hypothesis posits that large‐scale igneous activity during this period would have released significant amounts of CO_2_ into the atmosphere. However, there is no evidence of large igneous provinces (LIPs) coinciding with the third glaciation. Moreover, the accumulation of > 0.2 atm of CO_2_ in the atmosphere corresponds to the instantaneous introduction of over ~2.5 × 10^5^ Gt C into the atmosphere, which is comparable to maximum estimates of the total carbon emissions from one of the largest known massive volcanic events, that is, Ontong Java Nui (Roberge et al. 2004). Consequently, supplying this amount of CO_2_ to the ocean–atmosphere system solely via volcanic events is challenging. A theoretical study investigating the impact of LIPs on ocean–atmosphere redox dynamics (Watanabe et al. 2025) supports our conclusions that LIPs do not affect δ^34^S^b^_pyr_. Therefore, we propose that the pronounced negative δ^34^S^b^_pyr_ anomalies observed in the geological record (Luo et al. 2016; Izon et al. 2022) align well with the hypothesis that the third glaciation corresponded to a snowball Earth. It should be noted that most δ^34^S_pyr_ data are derived from bulk analyses, which may incorporate early diagenetic overprints or be influenced by sedimentary factors such as high deposition rates (Pasquier et al. 2021; Wang et al. 2021; Halevy et al. 2023; Zhu et al. 2025). Although these effects are unlikely to alter the large‐scale sulfur cycle trends simulated in this study, future comparisons between model results and grain‐scale sulfur isotope analyses will be valuable for refining these interpretations.

The disappearance and subsequent reappearance of S‐MIF signals across the Paleoproterozoic glaciations may record oscillations in atmospheric redox states following postglacial warming. Our model indicates that while postglacial oxidation could have transiently increased atmospheric O_2_ levels, the long‐term persistence of this oxidation depended on the supply of reductants (Figure 3). Under high reductant fluxes, the atmosphere could have reverted to a reducing state, leading to the reemergence of S‐MIF, whereas diminished reductant supply would have allowed stable atmospheric oxidation, resulting in the permanent loss of S‐MIF signatures.

Our calculations present several other testable predictions with implications for the geologic record. The results imply that large negative δ^34^S^b^_pyr_ anomalies are followed by large positive excursions in buried gypsum (> + 20‰). Therefore, our hypothesis can be tested by analyzing the sulfur isotope ratios of sulfate minerals after the third glaciation. If the oxidation event was temporary, the isotopic anomaly would decay in ~10^7^ years; however, if it was permanent, it would persist for > 10^7^ years. Although strict comparisons with the periods separating each glaciation are necessary, it may be possible to infer the consequences of an oxidation event by following the isotopic variation in gypsum. Notably, the simulation results also predicted a positive δ^34^S^b^_pyr_ anomaly (> + 5‰) after the negative anomaly (Figures 3D,J and 4D). In terms of the global S cycle, δ^34^S^b^_pyr_ is determined by the balance between the input (continental weathering and volcanic degassing) and fractionation via MSR; in a steady state, δ^34^S^b^_pyr_ does not exceed the sulfur isotope ratio of the input. Considering that the riverine δ^34^S should be close to the mantle value (~0‰) under pre‐GOE conditions, a positive δ^34^S^b^_pyr_ anomaly can only occur as a transient phenomenon associated with a large positive δ^34^S_sw_ anomaly. While the model predicts a subsequent positive δ^34^S^b^_pyr_ excursion following deglaciation, such a signal has not yet been clearly observed in existing geological records. The discovery of positive δ^34^S^b^_pyr_ anomalies in the geological record may provide evidence for large perturbations to the S cycle, although the formation of “superheavy” pyrite under sulfate‐limited conditions (Ries et al. 2009) provides a plausible alternative interpretation. Our simulations are based on the third Paleoproterozoic glaciation; however, they can be extended to other Paleoproterozoic glaciations. The snowball Earth would have resulted in a significant increase in atmospheric O_2_ and oceanic SO_4_^2−^ concentrations after deglaciation, producing large anomalies in the isotopic ratios recorded in sulfide and sulfate minerals. The first Paleoproterozoic glaciation represented by Makganyene diamictite is believed to have been a snowball Earth (Kirschvink et al. 2000). The Bruce Formation, which represents the second glaciation, is overlain by a carbonate layer possibly corresponding to cap carbonate, a characteristic sediment of a snowball Earth (Young 2002). The temporal increases in the O_2_ concentration after the first and second glaciations suggested by geological evidence (Figure 1) may have been caused by snowball Earth conditions. Our results imply that the distinctive δ^34^S anomaly in sulfide and sulfate, as depicted in Figures 3 and 4, may also be found in sediments deposited after the Makganyene and Bruce formations.

Our simulations do not yet fully account for all aspects of the existing geologic record. For example, while the results suggest that the third glaciation was a snowball Earth event, evidence such as low paleolatitude diamictites or cap carbonates, typically associated with snowball Earth, has not been identified for this glaciation. This absence of evidence in the geologic record poses a challenge that future research must address. Despite these limitations, this study highlights a close link between the climate and redox dynamics during the Paleoproterozoic. As more δ^34^S data on sulfides and sulfates become available, integrating this information with S‐MIF is expected to provide further insights into the redox dynamics during the Paleoproterozoic.

## 4 Conclusions

Our model analysis supports a link between climatic upheavals and redox dynamics during the Paleoproterozoic. A key conclusion is that the magnitude of O_2_ and SO_4_^2−^ accumulation and the δ^34^S anomalies in sulfide and sulfate can be correlated with the extent of climate and carbon cycle variability. Our results indicate that the negative sulfide δ^34^S^b^_pyr_ anomaly (< −25‰) observed after the third Paleoproterozoic glaciation can be formed following the climate recovery from intense greenhouse climate, which requires at least ~0.2 atm of atmospheric *p*CO_2_ to accumulate in the atmosphere; therefore, it is likely indicative of the occurrence of a snowball Earth. Our results predict that a similar SO_4_^2−^ increase and isotope anomaly should be observable after the first glaciation represented by the Makganyene diamictite, which is believed to indicate a snowball Earth.

Our second major conclusion concerns distinguishing between temporary and permanent oxidation events. Our model results suggest that while the magnitude of the redox variability is highly influenced by the magnitude of climate disturbances, whether the redox shift is a temporal or permanent event is not driven by the disturbance but rather by the boundary conditions. The boundary conditions should include the input rate of reductants, the rate of CO_2_ degassing, and terrestrial weatherability, the evolution of which triggered the GOE. Our results predict that permanent oxidation of the atmosphere leads to a sustained positive δ^34^S_sw_ anomaly over more than 10^7^ years, which would be recorded in deposited sulfate minerals. Accordingly, a combined analysis of S‐MIF and sulfate isotopic ratios will enable a comprehensive understanding of redox dynamics throughout the Paleoproterozoic glaciation.

## Conflicts of Interest

The authors declare no conflicts of interest.

## Supporting information


**Data S1:** gbi70040‐sup‐0001‐Supinfo.docx.

## Data Availability

The data that support the findings of this study are available from the corresponding author upon reasonable request. Code Availability: The source code of the biogeochemical model can be obtained from the corresponding author upon reasonable request.
