# Supplementary material for: Redox Dynamics of the Atmosphere and Oceans Induced by the Paleoproterozoic Snowball Earth Events
Source: Geobiology. 2025 Dec 26;23(6):e70040. doi: 10.1111/gbi.70040 (PMC12743051; doi:10.1111/gbi.70040)
Supplement: Supplementary file 1 — Data S1: gbi70040‐sup‐0001‐Supinfo.docx. [file GBI-23-e70040-s001.docx]

**Supplementary Material for**

Redox dynamics of the atmosphere and oceans induced by the Paleoproterozoic snowball Earth events

**This PDF file includes:**

Figures S1 to S7

Tables S1 to S3

References

# **Supplementary figures**

# **Figure S1.** Time evolution of selected variables in the nominal runs (permanent oxidation scenario). Color shades of lines indicate different initial values of atmospheric *p*CO_2_ (*p*CO_2_^init^) (same as Fig. 3). (A) partial pressure of atmospheric CO_2_; (B) partial pressure of atmospheric O_2_; (C) organic carbon flux (blue lines: sum of weathering and degassing fluxes, purple lines: burial flux); (D) pyrite flux (blue lines: sum of weathering and degassing fluxes, purple lines: burial flux); (E) partial pressure of atmospheric CH_4_; (F) phosphate concentration in deep ocean; (G) carbonate carbon flux (blue lines: sum of weathering and degassing fluxes, purple lines: burial flux); (H) gypsum flux (blue lines: sum of weathering and degassing fluxes, purple lines: burial flux); (I) global temperature; (J) sulfate concentration in deep ocean; (K) carbon isotope in the atmosphere and surface ocean; (L) sulfur isotope of seawater (δ^34^S_sw_, blue) and burying pyrite (δ ^34^S^b^_pyr_, purple).

**Figure S2.** The time evolution of selected variables in nominal runs varying with the initial value of atmospheric *p*CO_2_ (*p*CO_2_^init^) (similar to Fig. S1 but temporal oxidation scenario). (A) partial pressure of atmospheric CO_2_; (B) partial pressure of atmospheric O_2_; (C) organic carbon flux (blue lines: sum of weathering and degassing fluxes, purple lines: burial flux); (D) pyrite flux (blue lines: sum of weathering and degassing fluxes, purple lines: burial flux); (E) partial pressure of atmospheric CH_4_; (F) phosphate concentration in deep ocean; (G) carbonate carbon flux (blue lines: sum of weathering and degassing fluxes, purple lines: burial flux); (H) gypsum flux (blue lines: sum of weathering and degassing fluxes, purple lines: burial flux); (I) global temperature; (J) sulfate concentration in deep ocean; (K) carbon isotope in the atmosphere and surface ocean; (L) sulfur isotope of seawater (δ^34^S_sw_, blue) and burying pyrite (δ^34^S^b^_pyr_, purple).

**Figure S3.** Pairwise relationships of four varied constants (*p*CO_2_^init^, initial pyrite (PYR^init^), *K*_MSR_ and Δ_max_) and simulated maximum δ^34^S^b^_pyr_ (δ^34^S^b,max^_pyr_) of Monte Carlo simulations. Blue plots indicate runs with −35‰ < δ^34^S^b,min^_pyr_ < −25‰ (N = 1,945) and green plots indicate runs with −40‰ < δ^34^S^b,min^_pyr_ < −20‰ (N = 3,764).

**Figure S4.** Values of *K*_MSR_ and Δ_max_ in the subsampled runs with low initial *p*CO_2_ (*p*CO_2_^init^ < 0.2 atm and −35‰ < δ^34^S^b,min^_pyr_ < −25‰; colored plots; N = 65). The color bar indicates the initial value of *p*CO_2_ (*p*CO_2_^init^) in atm. The all accepted runs (−35‰ < δ^34^S^b,min^_pyr_ < −25‰; N = 1,945) are shown in filled gray plots. The black diamond plot indicates *K*_MSR_ and Δ_max_ values used in the nominal runs.

**Figure S5.** Compiled data of isotopic fractionation via microbial sulfate reduction (Δ_MSR_) as a function of sulfate concentration (filled circles). The data is based on (1–4) and references therein. Blue curves depict the dependence of Δ_MSR_ on sulfate concentration applied in our subsampled MC runs with low initial *p*CO_2_ (*p*CO_2_^init^ < 0.2 atm and −35‰ < δ^34^S^b,min^_pyr_ < −25‰; N = 65). Gray curves depict the dependence of Δ_MSR_ on sulfate concentration in all the subsampled MC runs (−35‰ < δ^34^S^b,min^_pyr_ < −25‰; N = 1,945). The dotted black curve is based on the values adopted in the nominal runs.

**Figure S6.** Output of subsampled runs (<−35‰ < δ^34^S^b,min^_pyr_ <−25‰) out of 10,000 Monte Carlo simulations. Similar to Fig. 4 but with fixed CP_sea_ (CP_sea_ = 60; N = 2,054) (A) Partial pressure of atmospheric CO_2_; (B) partial pressure of atmospheric O_2_; (C) oceanic sulfate concentration ([SO_4_^2−^]); and (D) sulfur isotopes of seawater (δ^34^S_sw_, blue) and buried pyrite (δ^34^S^b^_pyr_, pink). Black dotted lines indicate the output of the nominal run (Fig. 3) with *p*CO_2_^init^ = 0.7 atm.

**Figure S7.** Histograms of accepted runs out of 10,000 Monte Carlo simulations. Similar to Fig. 5 but with fixed CP_sea_ (CP_sea_ = 60). Counts of subsampled runs as functions of (A) *p*CO_2_^init^, (B) initial pyrite (PYR^init^), (C) *K*_MSR_, and (D) Δ_max_. Light gray histograms indicate runs with −40‰ < δ^34^S^b,min^_pyr_ < −20‰ (*N* = 3,928), and dark gray histograms indicate runs with −35‰ < δ^34^S^b,min^_pyr_ < −25‰ (*N* = 2,054). Counts of runs as functions of the δ^34^S^b,min^_pyr_ values and (E) *p*CO_2_^init^, (F) initial pyrite (PYR^init^), (G) *K*_MSR_, and (H) Δ_max_.

# **Supplementary tables**

**Table S1**. Mass balance equations

| Label | Reservoir | Mass balance equation (mol/yr) |
| --- | --- | --- |
| **A**_AS_ | Inorganic carbon in the atmosphere and surface ocean | $\frac{dA_{AS}}{dt}=F_{oxi\_C}+F_{esc}+F_{vcc}+F_{voc}+F_{wc}+F_{wo\_C}-F_{pp}-F_{bc}+F_{dgS\_C}+F_{cir\_C}$ |
| **A**_D_ | Inorganic carbon in the deep ocean | $\frac{dA_{D}}{dt}= F_{dgD\_C}-F_{cir\_C}$ |
| **O**_AS_ | Oxygen (O_2_) in the atmosphere and surface ocean | $\frac{dO_{AS}}{dt}=F_{oph}-F_{dgS\_O}-F_{voc}-F_{wo\_O}+2\left( F_{bpyr}-F_{vpyr}-F_{wpyr} \right)-F_{esc}-F_{oxi\_O}+F_{cir\_O}$ |
| **O**_D_ | O_2_ in the deep ocean | $\frac{dO_{D}}{dt}=-F_{dgD\_O}-F_{cir\_O}$ |
| **M**_AS_ | Methane (CH_4_) in the atmosphere and surface ocean | $\frac{dM_{AS}}{dt}=F_{dgS\_M}+F_{dgD\_M}+F_{wo\_M}-F_{esc}-F_{oxi\_M}$ |
| **Ca**_S_ | Dissolved calcium (Ca^2+^) in the surface ocean | $\frac{d{Ca}_{S}}{dt}=F_{ws}+F_{wc}-F_{bc}+F_{vgyp}+F_{wgyp}-F_{bgyp}+F_{cir\_Ca}$ |
| **Ca**_D_ | Ca^2+^ in the deep ocean | $\frac{d{Ca}_{D}}{dt}=-F_{cir\_Ca}$ |
| **P**_S_ | Dissolved phosphorus (PO_4_^3−^) in the surface ocean | $\frac{dP_{S}}{dt}=F_{rp}-F_{po\_P}+F_{cir\_P}$ |
| **P**_D_ | PO_4_^3−^ in the deep ocean | $\frac{dP_{D}}{dt}=F_{po\_P}-F_{bo\_P}-F_{cir\_P}$ |
| **Alk**_S_ | Alkalinity in the surface ocean | $\frac{d{Alk}_{S}}{dt}=2\left( F_{ws}+F_{wc}-F_{bc} \right)+F_{cir\_Alk}$ |
| **Alk**_D_ | Alkalinity in the deep ocean | $\frac{d{Alk}_{D}}{dt}=-F_{cir\_Alk}$ |
| **S**_S_ | Sulfate (SO_4_^2−^) in the surface ocean | $\frac{dS_{S}}{dt}=F_{vgyp}+F_{vpyr}+F_{wgyp}+F_{wpyr}-F_{bgyp}-F_{bpyr}+F_{cir\_S}$ |
| **S**_D_ | SO_4_^2−^ in the deep ocean | $\frac{dS_{D}}{dt}=-F_{cir\_S}$ |
| **C** | Continental inorganic carbon (CaCO_3_) | $\frac{dC}{dt}=F_{bc}-F_{vcc}-F_{wc}$ |
| **G** | Continental organic carbon | $\frac{dG}{dt}=F_{bo}-F_{voc}-F_{wo}$ |
| **GYP** | Continental gypsum (CaSO_4_) | $\frac{dGYP}{dt}=F_{bgyp}-F_{vgyp}-F_{wgyp}$ |
| **PYR** | Continental pyrite | $\frac{dPYR}{dt}=F_{bpyr}-F_{vpyr}-F_{wpyr}$ |

**Table S2.** Equations incorporated in the model (revised from Harada et al. 2015).

| Symbol | unit | Description | Equation/Value | Ref. |
| --- | --- | --- | --- | --- |
| *F_vcc_* | mol/yr | Volcanic degassing of CO_2_ from continental inorganic carbon | $F_{vcc}=F_{vcc}^{*}\frac{C}{C^{*}}$  *F_vcc_^*^* = 6.65 × 10^12^ mol/yr, *C^*^* = 2.1 × 10^21^ mol | (5) |
| *F_voc_* | mol/yr | Volcanic degassing of CO_2_ from continental organic carbon | $F_{voc}=F_{voc}^{*}\frac{G}{G^{*}}$  *F_voc_^*^* = 1.25 × 10^12^ mol/yr, *G^*^* = 1.25 × 10^21^ mol | (5) |
| *F_vgyp_* | mol/yr | Volcanic degassing from continental gypsum | $F_{vgyp}=F_{vgyp}^{*}\frac{GYP}{{GYP}^{*}}$  *F_vgyp_^*^* = 0.5 × 10^12^ mol/yr, *GYP^*^* = 150 × 10^18^ mol | (5) |
| *F_vpyr_* | mol/yr | Volcanic degassing from continental pyrite | $F_{vpyr}=F_{vpyr}^{*}\frac{PYR}{{PYR}^{*}}$  *F_vpyr_^*^* = 0.3 × 10^12^ mol/yr, *PYR^*^* = 250 × 10^18^ mol | (5) |
| *F_wgyp_* | mol/yr | Weathering of gypsum | $F_{wgyp}=F_{wgyp}^{*}\frac{GYP}{{GYP}^{*}}\frac{F_{wc}}{F_{wc}^{*}}$  *F_wgyp_^*^* = 1.6 × 10^12^ mol/yr, *F_wc_^*^* = 13.35 × 10^12^ mol/yr | (6) |
| *F_wpyr_* | mol/yr | Weathering of pyrite | $F_{wpyr}=F_{wpyr}^{*}f_{a}c_{pyrw}\left\{ \frac{\left( \frac{pO_{2}}{{pO}_{2}^{*}} \right)}{\left( \frac{pO_{2}}{{pO}_{2}^{*}} \right)+k_{pyrw}} \right\}\frac{PYR}{{PYR}^{*}}$  *F_wpyr_^*^* = 1.3 × 10^12^ mol/yr, *f_a_* = 0.48, *c_pyrw_* = 1.017, *k_pyrw_* = 0.017 | (7), this study |
| *F_bgyp_* | mol/yr | Burial of gypsum | $F_{bgyp}=F_{bgyp}^{*}\frac{\left[ Ca \right]}{\left[ Ca \right]^{*}}\frac{\left[ {SO}_{4} \right]}{\left[ {SO}_{4} \right]^{*}}$  *F^*^_bgyp_* = *F^*^_wgyp_* + *F^*^_vgy_* | (6) |
| *F_bpyr_* | mol/yr | Burial of pyrite | $F_{bpyr}=F_{bpyr}^{*}\frac{\left[ {SO}_{4} \right]}{\left[ {SO}_{4} \right]^{*}}\frac{{pO}_{2}^{*}}{pO_{2}}\frac{F_{bo}}{F_{bo}^{*}}$  *F^*^_bpyr_* = *F^*^_wpyr_* + *F^*^_vpyr_* | (6) |
| *F_wo_* | mol/yr | Weathering of continental organic carbon | $F_{wo}=F_{wo}^{*}f_{a}c_{orgw}\left\{ \frac{\left( \frac{pO_{2}}{{pO}_{2}^{*}} \right)}{\left( \frac{pO_{2}}{{pO}_{2}^{*}} \right)+k_{orgw}} \right\}\frac{G}{G^{*}}$  *F_wpyr_^*^* = 3.75 × 10^12^ mol/yr, *f_a_* = 0.48, *c_orgw_* = 1.334, *k_orgw_* = 0.33 | (7) |
| *F_bc_* | mol/yr | Precipitation of carbonate carbon | $F_{bc}=k_{carb}f_{a}\left( \Omega_{cal}-1 \right)^{ncal}$  *k_carb_* = 2.00 × 10^12^, *n_cal_* = 1.7  *f_a_*: Relative land fraction = 0.8 | (8) |
| *Δ_MSR_* | ‰ | Isotope fractionation via microbial sulfate reduction | $\Delta_{MSR}=\Delta_{max}\frac{\left[ {SO}_{4} \right]}{\left[ {SO}_{4} \right]+K_{MSR}}$  Δ_max_ = 40.0‰, *K*_MSR_ = 2 × 10^−4^ M | This study |
| *Δ_OPH_* | ‰ | Isotope fractionation via oxygenic photosynthesis | 25 (fixed) | This study |
| *F_bo_P_* | mol/yr | Phosphorus burial | $F_{bo\_P}=\frac{F_{bo}}{{CP}_{sea}}$ | This study |
| *CP_sea_* | - | Ratio of reactive P burial to organic carbon burial | ${CP}_{sea}=\frac{k_{anox} k_{oxic}}{\left( 1-anox \right)k_{anox}+anox k_{oxic}}$  *k_oxic_* = 60, *k_anoxic_* = 200 | (6), this study |
| *anox* | - | Degree of anoxia | $anox=max\left( 1-k_{1}\frac{pO_{2}}{{pO}_{2}^{*}}\frac{F_{pp}^{*}}{F_{pp}}, 0 \right)$  *k_1_* = 0.997527 | (5, 6) |

^*^Standard values prescribed in this model or the present values

**Table S3.** Equations incorporated the model (adopted from Harada et al. 2015).

| Symbol | Unit | Description | Equation / value |
| --- | --- | --- | --- |
| *F_wc_* | mol C/yr | Weathering of carbonate rocks | $F_{wc}=f_{a}f_{e}f_{b}F_{wc}^{*}$ |
| *F_ws_* | mol C/yr | Weathering of silicate rocks | $F_{ws}=f_{a}f_{e}f_{b}F_{ws}^{*}$  *F_ws_^*^* = 6.65 × 10^12^ mol/yr |
| *f_a_* | − | Relative land fraction (fixed) | 1.00 (modern)  0.48 (2.3 Ga) |
| *f_e_* | − | Relative biological activity of soil (fixed) | 1.00 (modern)  0.25 (2.3 Ga) |
| *f_b_* | − | Kinetic control on weathering | $f_{b}=\left( \frac{{pCO}_{2}}{{pCO}_{2}^{*}} \right)^{n}\frac{exp\left( -\frac{E}{RT} \right)}{exp\left( -\frac{E}{RT^{*}} \right)}$ |
| *E* | kcal mol^−1^ | Activation energy | 15 |
| *R* | kcal K^−1^  mol^−1^ | Gas constant | 1.99 × 10^−3^ |
| *n* | - | Exponent of *p*CO_2_ dependency | 0.3 |
| *F_rp_* | mol P/yr | Phosphorus river input | $F_{rp}=\frac{F_{ws}+F_{wc}}{F_{ws}^{*}+F_{wc}^{*}} F_{rp}^{*}$  *F_rp_^*^*: prescribed to balance with the steady state *F_bo_P_* value |
| *F_pp_* | mol C/yr | Net primary productivity | $F_{pp}=\frac{F_{po}}{f_{export}}$ |
| *f_export_* | − | Fraction of organic matter exported from surface to deep water | 0.0410 |
| *F_po_* | mol C/yr | Export production | $F_{po}=R_{cp}\left[ \mathrm{PO}_{4} \right]_{S}\frac{\left[ \mathrm{PO}_{4} \right]_{S}}{\left[ \mathrm{PO}_{4} \right]_{S}+\gamma_{p}}$ |
| *F_po_P_* | mol P/yr | Phosphorus uptake by export production | $F_{po\_P}=\frac{F_{po}}{R_{cp}}$ |
| *R_cp_* | − | C/P ratio | 106 |
| *γ_p_* | mol/l | Half saturation constant | 1.00 × 10^−9^ |
| *F_bo_* | mol C/yr | Burial of organic carbon | $F_{bo}=f_{burial}F_{po}$ |
| *f_burial_* | − | Fraction of organic matter buries into sediment | 0.0325 |
| *F_oph_* | mol C/yr | oxygenic photosynthesis | *F_oph_* = *F_pp_* − *F_red_* |
| *F_red_* | mol O_2_/yr | Excess input of reductant from Earth’s interior (fixed) | 0.075 × 10^12^  0.3 × 10^12^ |
| *F_dgS_M_* | mol C/yr | CH_4_ production by decomposition of particulate organic matter in the surface water | $F_{dgS\_M}=\frac{1}{2}\Phi_{\left( \text{O}_{\text{2}} \right)S}\left( 1-f \right)F_{pp}$ |
| *F_dgD_M_* | mol C/yr | CH_4_ production by decomposition of particulate organic matter in the deep water | $F_{dgD\_M}=\frac{1}{2}\Phi_{\left( \text{O}_{\text{2}} \right)D}\left( 1-\alpha\right)F_{po}$ |
| *F_dgS_C_* | mol C/yr | CO_2_ production by decomposition of particulate organic matter in the surface water | $F_{dgS\_C}=\left\{ \gamma_{\left( \text{O}_{\text{2}} \right)S}+\frac{1}{2}\left( 1+\delta_{S} \right)\left( 1-\gamma_{S} \right) \right\}\left( 1-f \right)F_{pp}$ |
| *F_dgD_C_* | mol/yr | CO_2_ production by decomposition of particulate organic matter in the deep water | $F_{dgD\_C}=\left\{ \gamma_{\left( \text{O}_{\text{2}} \right)D}+\frac{1}{2}\left( 1+\delta_{D} \right)\left( 1-\gamma_{D} \right) \right\}\left( 1-f \right)F_{po}$ |
| *F_dgS_O_* | mol/yr | O_2_ consumption by decomposition of particulate organic matter in the surface water | $F_{dgS\_O}=\left( 1-\Phi_{\left( \text{O}_{\text{2}} \right)S} \right)\left( 1-f \right)F_{pp}$ |
| *F_dgD_O_* | mol/yr | O_2_ consumption by decomposition of particulate organic matter in the deep water | $F_{dgD\_O}=\left( 1-\Phi_{\left( \text{O}_{\text{2}} \right)D} \right)\left( 1-\alpha\right)F_{po}$ |
| *F_cir_X_* | mol/yr | Exchange of dissolved component **X** between surface ocean and deep ocean | $F_{cir\_X}=\left( {[X]}_{D}-{[X]}_{S} \right)W$ |
| *W* | l/yr | − | 5.99 × 10^17^ |
| *F_oxi_M_* | mol/yr | CH_4_ consumption by photochemical oxidation of CH_4_ | $F_{oxi\_M}=\frac{1}{2}M^{0.7}\Psi_{\left( \text{O}_{\text{2}} \right)S}$ |
| *F_oxi_O_* | mol/yr | O_2_ consumption by photochemical oxidation of CH_4_ | $F_{oxi\_O}=M^{0.7}\Psi_{\left( \text{O}_{\text{2}} \right)S}$ |
| Ψ_(O2)S_ |  | Oxidation parameter | ${10}^{a_{1}\psi^{4}+a_{2}\psi^{3}+a_{3}\psi^{2}+a_{4}\psi+a_{5}}$, $\psi=\text{log}\text{O}_{\text{AS}}$ |
| *a_1_* | − | − | 0.003006 |
| *a_2_* | − | − | −0.1655 |
| *a_3_* | − | − | 3.2305 |
| *a_4_* | − | − | −25.8343 |
| *a_5_* | − | − | 71.5398 |
| *F_esc_H_* | mol/yr | Hydrogen escape | $F_{esc\_H}=sM$ |
| *s* | yr^−1^ | Proportion constant | 3.70 × 10^−5^ |
| *γ*_(O2)_*_S_* | − | Fraction of organic matter decomposed by aerobic respiration in surface water | $\gamma_{\left( \text{O}_{\text{2}} \right)S}=\frac{O_{AS}}{O_{AS}+d_{\gamma}}=\frac{\left[ \text{O}_{\text{2}} \right]_{S}}{\left[ \text{O}_{\text{2}} \right]_{S}+d_{\gamma}^{'}}$ |
| *γ*_(O2)_*_D_* | − | Fraction of organic matter decomposed by aerobic respiration in deep water | $\gamma_{\left( \text{O}_{\text{2}} \right)D}=\frac{\left[ \text{O}_{\text{2}} \right]_{D}}{\left[ \text{O}_{\text{2}} \right]_{D}+d_{\gamma}^{'}}$ |
| *δ*_(O2)_*_S_* | − | Fraction of methane decomposed by methanotrophs in atmosphere and surface water | $\delta_{\left( \text{O}_{\text{2}} \right)S}=\frac{O_{AS}}{O_{AS}+d_{\delta}}=\frac{\left[ \text{O}_{\text{2}} \right]_{S}}{\left[ \text{O}_{\text{2}} \right]_{S}+d_{\delta}^{'}}$ |
| *δ*_(O2)_*_D_* | − | Fraction of methane decomposed by methanotrophs in atmosphere and deep water | $\delta_{\left( \text{O}_{\text{2}} \right)D}=\frac{\left[ \text{O}_{\text{2}} \right]_{D}}{\left[ \text{O}_{\text{2}} \right]_{D}+d_{\delta}^{'}}$ |
| *d_γ_'* | mol/l | Half saturation constant | 1.00 × 10^−5^ |
| *d_δ_'* | mol/l | Half saturation constant | 2.00 × 10^−6^ |
| Φ_(O2)S_ | − | Fraction of organic matter anaerobically decomposed in atmosphere and surface ocean | $\Phi_{\left( \text{O}_{\text{2}} \right)S}=\left( 1-\gamma_{\left( \text{O}_{\text{2}} \right)S} \right)\left( 1-\delta_{\left( \text{O}_{\text{2}} \right)S} \right)$ |
| Φ_(O2)D_ | − | Fraction of organic matter anaerobically decomposed in atmosphere and deep ocean | $\Phi_{\left( \text{O}_{\text{2}} \right)D}=\left( 1-\gamma_{\left( \text{O}_{\text{2}} \right)D} \right)\left( 1-\delta_{\left( \text{O}_{\text{2}} \right)D} \right)$ |
| *V_S_* | L | Volume of the surface ocean | 0.05 × 10^21^ |
| *V_D_* | L | Volume of the deep ocean | 1.37 × 10^21^ |

**References**

1. K. S. Habicht, M. Gade, B. Thamdrup, P. Berg, D. E. Canfield, Calibration of sulfate levels in the archean ocean. *Science* **298**, 2372–2374 (2002).

2. K. S. Habicht, L. Salling, B. Thamdrup, D. E. Canfield, Effect of low sulfate concentrations on lactate oxidation and isotope fractionation during sulfate reduction by Archaeoglobus fulgidus strain Z. *Appl. Environ. Microbiol.* **71**, 3770–3777 (2005).

3. A. S. Bradley, *et al.*, Patterns of sulfur isotope fractionation during microbial sulfate reduction. *Geobiology* **14**, 91–101 (2016).

4. M. S. Sim, *et al.*, What controls the sulfur isotope fractionation during dissimilatory sulfate reduction? *ACS Environ. Au* **3**, 76–86 (2023).

5. T. M. Lenton, S. J. Daines, B. J. W. Mills, COPSE reloaded: An improved model of biogeochemical cycling over Phanerozoic time. *Earth Sci. Rev.* **178**, 1–28 (2018).

6. N. M. Bergman, T. M. Lenton, A. J. Watson, COPSE: A new model of biogeochemical cycling over Phanaerozoic time. *Am J Sci* **304**, 397–437 (2004).

7. K. Ozaki, D. B. Cole, C. T. Reinhard, E. Tajika, V, Canops-Grb, A new Earth system model for simulating the evolution of ocean-atmosphere chemistry over geologic timescales. *Geosci Model Dev* **15**, 7593–7639 (2022).

8. J. Krissansen-Totton, G. N. Arney, D. C. Catling, Constraining the climate and ocean pH of the early Earth with a geological carbon cycle model. *Proc. Natl. Acad. Sci. U. S. A.* **115**, 4105–4110 (2018).
